# ATF3-mediated inhibition of Trem2 by *Toxoplasma gondii* contributes to adverse pregnancy outcomes

**DOI:** 10.1186/s13071-025-06894-w

**Published:** 2025-07-01

**Authors:** Xiaoyu Geng, Tiankun Yao, Xinyue Wang, Jianan Wang, Tongrui Zhang, Tianmei Qian, Chun Liu, Jinling Chen

**Affiliations:** 1 Department of Pathogen Biology, School of Medicine, Nantong University, 19 Qixiu Road, Nantong, 226001 Jiangsu People’s Republic of China; 2 Laboratory Animal Center, Nantong University, 19 Qixiu Road, Nantong, 226001 Jiangsu People’s Republic of China; 3 Nanjing Medical University, Nanjing, 211166 Jiangsu People’s Republic of China; 4 Engineering Research Center of Integration and Application of Digital Learning Technology, Ministry of Education, Beijing, 100034 People’s Republic of China; 5 NMPA Key Laboratory for Research and Evaluation of Tissue Engineering Technology Products, Nantong University, Nantong, 226001 Jiangsu People’s Republic of China

**Keywords:** *Toxoplasma gondii*, Adverse pregnancy outcomes, Macrophages, *Trem2 *promoter, *ATF3*

## Abstract

**Background:**

Approximately one in three people worldwide have been exposed to *Toxoplasma gondii* (*T. gondii*). Primary infection with *T. gondii* during pregnancy can cause severe complications. Our previous study demonstrated that deficiency of triggering receptor expressed on myeloid cells 2 (*Trem2*) exacerbates pregnancy-related complications in *T. gondii*-infected mice. However, understanding the mechanisms by which *T. gondii* modulates Trem2 expression in macrophages remains an unmet challenge.

**Methods:**

A mouse pregnancy model of *T. gondii* infection and an in vitro cellular stimulation model using soluble *T. gondii* antigens (s*Tg*Ag) were used to assess Trem2 expression. Recombinant plasmids containing the full-length *Trem2* promoter were constructed to evaluate the effect of s*Tg*Ag on promoter activity, followed by the construction of truncated promoter plasmids to identify key regulatory regions. Transcription factors potentially binding to the* Trem2* promoter were predicted using PROMO and JASPAR, with ATF3 identified as responsive to s*Tg*Ag stimulation via western blot analysis. The binding of *ATF3* to the *Trem2* promoter was validated by chromatin immunoprecipitation (ChIP) assays. Finally, *ATF3* knockdown experiments were performed to determine its role in mediating the inhibitory effect of s*Tg*Ag on *Trem2* expression.

**Results:**

*T. gondii* significantly suppressed *Trem2* expression in both mouse placentas and cellular models, with truncated promoter assays identifying key regulatory regions of the *Trem2* promoter inhibited by s*Tg*Ag. *ATF3* was identified as a transcription factor responsive to s*Tg*Ag stimulation, which bound to the *Trem2* promoter. Importantly, knockdown of* ATF3* restored *Trem2* expression, demonstrating its critical role in mediating the inhibitory effect of s*Tg*Ag.

**Conclusions:**

We identified that s*Tg*Ag may target and inhibit *Trem2* expression through the transcription factor *ATF3*, and inhibition of *ATF3* activity may help maintain *Trem2* expression in macrophages, providing a potential therapeutic approach to avert negative effects on pregnancy related to *T. gondii* infection.

**Graphical Abstract:**

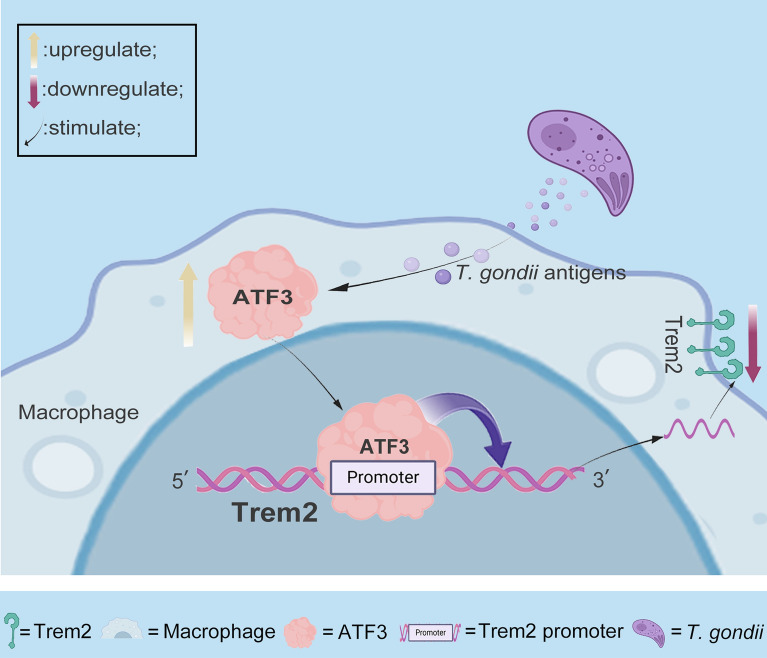

**Supplementary Information:**

The online version contains supplementary material available at 10.1186/s13071-025-06894-w.

## Background

*T. gondii*, an opportunistic pathogenic protozoon, infects a variety of warm-blooded animals [1, 2]. As a well-established etiological agent in TORCH syndrome (toxoplasmosis, cytomegalovirus, among others), *T. gondii* infection can cause dysregulation of maternal immune homeostasis, particularly in early pregnancy, thereby increasing the risk of adverse pregnancy outcomes (APOs), including miscarriage and fetal teratogenesis [3]. Increasing evidence suggests that APOs related to *T. gondii* infection occur primarily due to an abnormal immune microenvironment at the maternal–fetal interface [4, 5]. The interface, which consists of trophoblastic cells and immune cells, is pivotal for the establishment and maintenance of normal pregnancy [6, 7]. Several studies have shown the crucial role of decidual macrophages (dMφ) in maintaining immune balance during pregnancy [8, 9]. During early infection, dMφ secretes pro-angiogenic factors and cytokines that can either promote inflammation (e.g., tumor necrosis factor [TNF]-α and interleukin [IL]-1β) or suppress it (e.g., transforming growth factor [TGF]-β and IL-10). This balance facilitates trophoblast invasion and placental development while enhancing defenses against pathogenic threats [10]. In addition, *T. gondii* infection alters the quantity and function of dMφ, promoting M1 macrophage polarization, which disrupts the immunosuppressive microenvironment at the maternal–fetal interface and may result in APOs [11, 12]. In studies investigating how *T. gondii* infection leads to APOs, functional molecules such as T-cell immunoglobulin and mucin-domain containing molecule 3 (Tim-3) and B7 homolog 4 (B7-H4) have been explored [13]. The downregulation of B7-H4 increases iNOS and TNF-α expression via the JAK2/STAT1 pathway, while reduced Tim-3 activates the PI3K-AKT and JAK-STAT pathways, enhancing perforin and IL-10 production [14]. However, the role of *Trem2* in this context remains unexplored, prompting us to examine its potential involvement in *T. gondii* infection.

Recently, we identified *Trem2* as a critical regulator of dMφ in the response to *T. gondii* invasion [15]. *Trem2*, a transmembrane protein containing a V-immunoglobulin structural domain, relies on electrostatic interactions with DNAX-activating protein 12 (DAP12) for intracellular activity [16, 17]. Previous studies have reported that *Trem2* suppresses CXC chemokine ligand 3 (CXCL3) expression by targeting the phosphatidylinositol 3-kinase (PI3K)-AKT pathway and promotes macrophage polarization from M1 to M2, thereby attenuating inflammatory responses [18, 19]. Moreover, experimental evidence indicates that *Trem2* serves as a key marker for macrophage activation and is among the 12 cluster-defining genes associated with endosomal and lysosomal systems in cluster 0 macrophages. *Trem2* is proposed to facilitate phagocytosis and clearance of abnormal cellular debris, a function critical for maintaining maternal–fetal tolerance [20]. Recent studies have reported that in a normal pregnancy model, the expression of *Trem2* on dMφ was significantly diminished following *T. gondii* infection. In addition, deficiency of *Trem2* exacerbated the APOs associated with *T. gondii* in mice [15]. These results point to the likelihood that *Trem2* could be key in protecting against the harmful effects of *T. gondii* during pregnancy. However, the regulation of *Trem2 *gene expression remains unknown, especially at the promoter level, as prior studies have predominantly emphasized its signaling pathways and functional mechanisms [21]. In this study, we aimed to fill this gap by focusing on the regulatory mechanisms of the *Trem2* promoter. By analyzing the *Trem2* promoter region, we identified several key transcription factors binding sites and explored the effect of *ATF3* on *Trem2 *promoter activity.

Promoters are usually located upstream of the 5′ end of structural genes and can bind to RNA polymerase and transcription factors to regulate and control transcription initiation [22]. The activity of promoters is closely related to the interaction of transcription factors (TFs) [23], which may stimulate or inhibit gene expression in response to external stimuli on the basis of environmental changes perceived by the organism [24]. *ATF3*, belonging to the ATF/CREB transcription factor family, is a transcription factor that responds to stress and is essential for the regulation of metabolism, immune responses, and tumor formation [25]. Evidence suggests that blockade and silencing of *ATF3* or AP1S2 significantly inhibits inflammatory bowel disease via CD14^+^-derived M1-like macrophage polarization and pro-inflammatory cytokine production [26]. Notably, our study demonstrates that s*Tg*Ag markedly enhances ATF3 expression, which binds to the *Trem2* promoter. These findings offer a novel understanding of the mechanisms behind *Trem2* molecules in leading to APOs mediated by *T. gondii* through its effects on macrophage function.

In this investigation, we demonstrated that *ATF3* acts as a key transcription regulator essential for *Trem2* expression. In Raw264.7 mouse macrophages subjected to s*Tg*Ag stimulation, both *Trem2* expression and its promoter activity were significantly downregulated, concurrently accompanied by an upregulation of the transcription factor *ATF3*. These results indicate that ATF3 negatively regulates *Trem2* expression. Furthermore, this finding implies that inhibiting *ATF3* activity may sustain *Trem2* expression in macrophages, providing a potential therapeutic strategy to prevent and mitigate *T. gondii*-associated pregnancy complications. Therefore, more exploration of the mechanism of *ATF3* interaction with the *Trem2* promoter may have important clinical implications.

## Methods

### Animals

C57BL/6 mice were obtained and bred at the Nantong University Laboratory Animal Center. Mice were mated in a 1:2 male-to-female ratio overnight. Females were checked before 08:00 the following day for a seminal plug, marking it as gestation day 0.5 (E0.5) [27]. Pregnant mice were then divided into two categories: a normal pregnancy group (PBS) and a pregnancy group infected with *T. gondii* (*T. gondii*). At E8.5, pregnant mice were injected intraperitoneally with 300 tachyzoites of *T. gondii* (RH strain) and euthanized using CO_2_ asphyxiation at E17.5. Mouse fetuses were photographed and weighed and all mouse placentas were collected for subsequent experiments. Fetal mouse development was evaluated on the basis of fetal weight and size, which was measured and depicted by the crown-rump length (CRL) and the occipital-frontal diameter (OFD) of the fetal head (CRL × OFD) [28]. All mouse procedures received approval from the Animal Care and Use Committee of Nantong University (approval number: P20230302-013).

### Isolation of soluble antigens from *T. gondii*

The *T. gondii* (RH strain) used in the research was generously provided by Y. Wang from Nanjing Medical University. s*Tg*Ag was extracted from the tachyzoites according to the methodologies outlined by Qiu et al. [29]. In brief, tachyzoites were isolated using a 3 μm pore-size track-etched membrane (Shanghai Neng think Filtration Technology, Shanghai, China) for the removal of host cell debris. After confirming tachyzoite viability (> 95%) using trypan blue assay, around 1 × 10^8^ tachyzoites of *T. gondii* were cultivated in serum-free RPMI-1640 medium (Gibco, Grand Island, NY, USA) for a duration of 3 h. The culture supernatants were centrifuged and concentrated using Amicon® Ultra-15 centrifugal filters (Merck Millipore, Germany, Darmstadt). Subsequently, an endotoxin removal kit (Thermo Fisher Scientific, Massachusetts, USA) was employed to eliminate the endotoxins from s*Tg*Ag.

### Cells and treatments

The Raw264.7 cell line, a murine macrophage cell line derived from an Abelson leukemia virus-induced tumor, was obtained from the Cell Resource Center at the Shanghai Institute of Life Science (Shanghai, China). Raw264.7 cells (a maximum of 8 passages) were cultured in Dulbecco’s modified Eagle medium (DMEM; Thermo) supplemented with 10% heat-inactivated fetal bovine serum (FBS; ExCell Bio, Jiangsu, China) and 1% penicillin–streptomycin (Thermo) at 37 °C in a 5% CO_2_ humidified atmosphere. Raw264.7 cells were treated with s*Tg*Ag (5 µg/ml) for either 48 h for protein detection or 24 h for gene expression analysis. All cells were maintained in a humidified environment with 5% CO_2_ at 37 ℃. Upon reaching 70–80% confluence, Raw264.7 cells were passaged at a 1:2 ratio. All cell experiments were performed with three biological and technical replicates.

### Western blot

Fresh mouse placentas or cells were washed with PBS, and total protein was extracted using ice-cold RIPA lysis buffer supplemented with 1% phenylmethanesulfonyl fluoride (PMSF) and 1% phosphatase inhibitor cocktail (RIPA: PMSF: phosphatase inhibitor = 100:1:1). Protein concentration was quantified using an Ultra-micro-Spectrophotometer (One drop OD-1000, Nanjing, China). Equal amounts of protein (20 μg per lane) were separated by SDS-PAGE (8–15%) and electrically transferred onto polyvinylidene difluoride membranes (PVDF, Merck). Following a blocking step with skim milk, the PVDF membrane was cultured with primary antibody in Tris-buffered saline with Tween-20 (TBST), and subsequently subjected to incubation with the appropriate horseradish peroxidase-conjugated secondary antibodies. PVDF membranes were analyzed through enhanced chemiluminescence (ECL, Meilunbio, Dalian, China). The following antibodies were applied in the study: *Trem2* antibody (1:3000, mouse monoclonal; R&D Systems, Minnesota, USA, no. AF1729), recombinant anti-ATF3 antibody (1:1000, rabbit monoclonal; Abcam, Cambridge, UK, EPR22610-19, no. ab254268), STAT6 (D3H4) mAb (1:1000, rabbit monoclonal; CST, Danvers, MA, USA, D3H4, no. 5397), GAPDH antibody (1:50,000, mouse monoclonal; 1E6D9, no. 60004-1-Ig), HRP-conjugated goat anti-mouse IgG (H + L) (1:5000, mouse monoclonal; no. SA00001-1) ordered from Proteintech (Rosemont, IL, USA), HRP-conjugated goat anti-rabbit IgG (H + L) (1:5000, rabbit monoclonal; Biosharp, Jiangsu, China, no. BL003A).

### Hematoxylin–eosin (HE) staining

Mouse placenta from E17.5 mice was fixed in 4% paraformaldehyde (PFA, biosharp, BL539A) for 24 h at 4 ℃, dehydrated in graded ethanol (70% → 100%, 1 h/step), cleared in xylene (2 × 30 min), and paraffin-embedded. Sections (5 µm) were deparaffinized in xylene (2 × 10 min), rehydrated (100% → 70% ethanol, 2 min/step), stained with Mayer’s hematoxylin (5 min; Sigma, Aldrich, USA, no. MHS16) and eosin (2 min; Sigma, no. 318906), dehydrated (70% → 100% ethanol, 30 s/step), cleared in xylene (2 × 5 min), and mounted with neutral gum (Beyotime, Shanghai, China, no. C0173). Images were acquired using a Leica DM5000 B microscope (Leica Biosystems, Wetzlar, Germany).

### RNA isolation and quantitative RT-PCR analysis

Total RNA was extracted from Raw264.7 cells utilizing Trizol Reagent (Invitrogen, CA, USA) following the manufacturer’s protocol, including chloroform phase separation (10 min), isopropanol precipitation (10 min), and 75% ethanol washes. cDNA was synthesized from 1 μg RNA using PrimeScript^™^ RT Reagent Kit (Takara, Shiga, Japan, no. RR037A; 37 ℃ 15 min, 85 ℃ 5 s). qPCR was performed with TB Green^™^ Premix Ex Taq^™^ II (Takara no. RR820A) on a StepOnePlus Real-Time PCR System (Applied Biosystems, Waltham, MA, USA) using 0.4 µl primers per 20 µl reaction (95 ℃ 30 s; 40 cycles of 95 ℃ 5 s, 62 ℃ 30 s, and 72 ℃ 30 s). *GAPDH*-normalized data were analyzed by ^ΔΔ^Ct method. Primer sequences were as follows: (1) *Trem2* forward: 5′-TGCTGGAACCGTCACCATCAC-3′; reverse: 5′-ACTTGGGCACCCTCGAAACTC-3′; (2) GAPDH forward: 5′-TGGAAAGCTGTGGCGTGAT-3′; reverse: 5′-TGCTTCACCACCTTCTTGAT-3′.

### Dual-luciferase reporter assay

Cells were placed into 24-well plates at a density of 1 × 10^5^ cells per well and incubated overnight. The luciferase reporter plasmid, together with the *Renilla* luciferase plasmid (pRL-TK), were co-transfected into cells utilizing the jetPRIME transfection reagent (Polyplus, Strasbourg, French). At 6 h post-transfection, Raw264.7 cells were treated with s*Tg*Ag (5 µg/ml) for 24 h. Firefly and *Renilla* luciferase activities were assessed by using the Dual-Luciferase Reporter Assay System (Promega, Madison, WI, USA). Recombinant plasmids containing the full-length *Trem2 *promoter, truncated promoters, and mutation sequences in the ATF3 binding site, were constructed from the pLenti-AR-minP-Luc-PGK-Puro vector (empty vector) and were designated as pLenti-Trem2-minP-Luc-PGK-Puro (p-Trem2), p-Trem2-A (−1542 to +200), p-Trem2-B (−851 to +200), p-Trem2-C (−448 to +200), and p-Trem2-A-Mut.

### Immunofluorescence

The four-color multiplex fluorescence immunohistochemical staining kit (Absin, Shanghai, China) was employed for immunofluorescence staining. Cells were initially fixed using 4% paraformaldehyde (PFA, biosharp, 15 min), which was followed by a permeabilization step lasting 15 min and blocked with 5% BSA (20 min). The samples were subsequently incubated with the recombinant anti-ATF3 antibody (1:100, rabbit monoclonal; Abcam, EPR22610-19, no. ab254268) and recombinant anti-Trem2 antibody (1:500; rabbit monoclonal; Abcam, EPR26210-1, no. ab305103), before being treated with the corresponding secondary antibody. The nuclei were stained and an anti-fluorescence quenching sealer was applied to enhance stability. Finally, it was photographed utilizing a confocal laser scanning microscope (Olympus, Hachioji, Japan).

### RNA interference

All small interference RNAs (siRNAs) targeting *ATF3*, as well as the negative control small interference RNA (siNC), were ordered from Genepharma (Jiangsu, China). Raw264.7 cells were transfected with the indicated siRNAs using INTERFERin Reagent (Polyplus, Strasbourg, French). Following a 6 h incubation period, the transfected-Raw264.7 cells were stimulated for 48 h with s*Tg*Ag (5 µg/ml) or medium only. The primers for *ATF3*: forward: 5′-GCAUCCUUUGUCUCACCAATT-3′; reverse: 5′-UUGGUGAGACAAAGGAUGCTT-3′.

### Chromatin immunoprecipitation

Chromatin immunoprecipitation (ChIP) experiments were conducted utilizing the Simple ChIP Enzymatic Chromatin IP Kit (CST). Following formaldehyde fixation of cellular structures, chromatin was subjected to fragmentation via *Micrococcus* nuclease and sonication, yielding chromatin fragments encompassing 1–5 nucleosomes. The samples were then incubated overnight with the positive control histone H3 antibody, the ATF3 antibody (5 µg per 25 µg of chromatin), and the negative control normal rabbit IgG antibody, to generate antibody–protein–DNA complexes. Protein G Plus beads were incorporated into the complexes alongside the antibodies and the corresponding control IgG for immunoprecipitation. Following the IP procedure, the Protein G Plus beads were washed with buffers of varying ionic strengths. The protein–DNA complexes were then eluted from the beads, and the DNA was purified using DNA purification centrifugal columns. Finally, the purified DNA was validated for analysis via PCR. The primers for *Trem2*: (1) forward: 5′-GTCCTCTAGAACTCCAGGAAATGC-3′; reverse: 5′-TGGTTGCTGGGATTTGAACTCG-3′; (2) forward: 5′-AATTTTAAAAAACGGGGGGAGGGG-3′; reverse: 5′-CTCCAGAAGAGAGCATCAGATTT-3′.

### Statistical analyses

All experiments were conducted with a minimum of three biological replicates. Data were expressed as means ± SD. Statistical analyses were conducted using GraphPad Prism 9.0 software (La Jolla, CA, USA). A two-tailed independent samples *t*-test was utilized to analyze variations between two groups, while one-way analysis of variance (ANOVA) with Tukey’s multiple comparisons test and two-way ANOVA with Sidak correction were utilized to analyze distinctions across multiple groups. A *P*-value of less than 0.05 was deemed to indicate a significant difference.

## Results

### Soluble *T. gondii* antigens inhibit *Trem2* expression in vitro

**Fig. 1:**
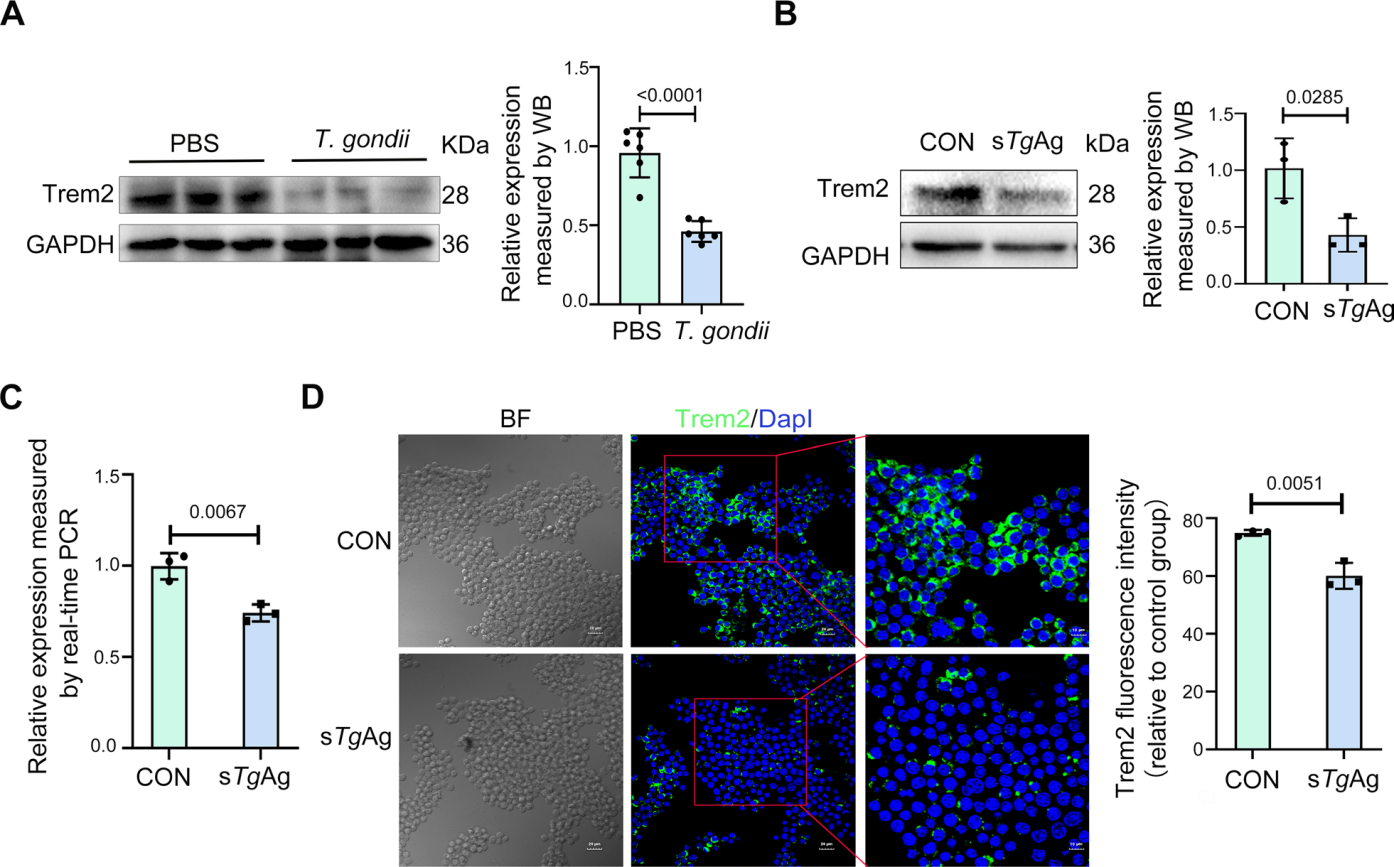
Soluble *T. gondii* antigens inhibit *Trem2* expression in vitro. **A** *Trem2* expression in the placentas of *T. gondii*-infected and normal pregnancy (PBS) mice were assessed using western blotting. Each data point corresponds to the placenta isolated from one pregnant mouse (*n* = 6 mice). **B** The expression of *Trem2* was assessed utilizing western blotting after Raw264.7 cells were exposed to s*Tg*Ag (5 µg/ml) for 48 h. The analysis results were obtained using Image J. **C** The mRNA level of *Trem2* in Raw264.7 cells was assessed using real-time quantitative PCR following the stimulation of Raw264.7 cells with 5 µg/ml of s*Tg*Ag for 24 h, using* GAPDH *as an internal control. **D** Immunostaining of *Trem2* was performed in Raw264.7 cells exposed to s*Tg*Ag (5 µg/ml) for 48 h. Scale bars, 20 µm. Two-tailed unpaired Student’s *t*-tests used for all data; *n* = 3 independent experiments; mean ± SD; s*Tg*Ag, soluble *T. gondii* antigens; CON, the untreated control group. Quantifications normalized to GAPDH for western blot

A *T. gondii*-infected mouse pregnancy model was used through intraperitoneal injection of *T. gondii* tachyzoites at E8.5, which resulted in increased fetal resorption and mortality observed at E17.5 (Fig. S1A). Measurement of fetal size and weight indicated that *T. gondii* infection likewise led to constrained intrauterine fetal development (Fig. S1B and C). We investigated the effects of *T. gondii* infection on mouse placentas utilizing HE staining. Results showed significant necrosis and hemorrhage in the decidual zone (De) of infected placenta, suggesting that *T. gondii* induced structural disruption of the placenta and impaired material exchange (Fig. S1D). In addition, protein blotting assays revealed a substantial reduction in *Trem2* protein expression within the placental tissues of infected mice compared with their uninfected counterparts (Fig. 1A). These results suggest that *T. gondii* infection can induce APOs, potentially linked to the downregulation of *Trem2* expression.

The APOs induced by *T. gondii* infection are primarily associated with the disruption of immune tolerance, rather than the replication of *T. gondii* itself [30]. s*Tg*Ag are often utilized to investigate the mechanisms of maternal–fetal immune imbalance induced by *T. gondii* [29]. Within this study, the relationship between s*Tg*Ag and *Trem2* was examined by culturing macrophage (Raw264.7) cells in vitro and stimulating them with 5 µg/ml of s*Tg*Ag for 48 h. The level of *Trem2* was subsequently assessed using both protein immunoblotting assays and RT-qPCR. We discovered that s*Tg*Ag reduced the protein and gene level of *Trem2* (Fig. 1B and C). In addition, immunostaining with the *Trem2* antibody revealed a significant decrease in *Trem2* expression in Raw264.7 cells stimulated with s*Tg*Ag (Fig. 1D). These data suggest that s*Tg*Ag can inhibit the expression of *Trem2* in vitro.

### Soluble *T. gondii* antigens inhibit the activity of the *Trem2* promoter

**Fig. 2:**
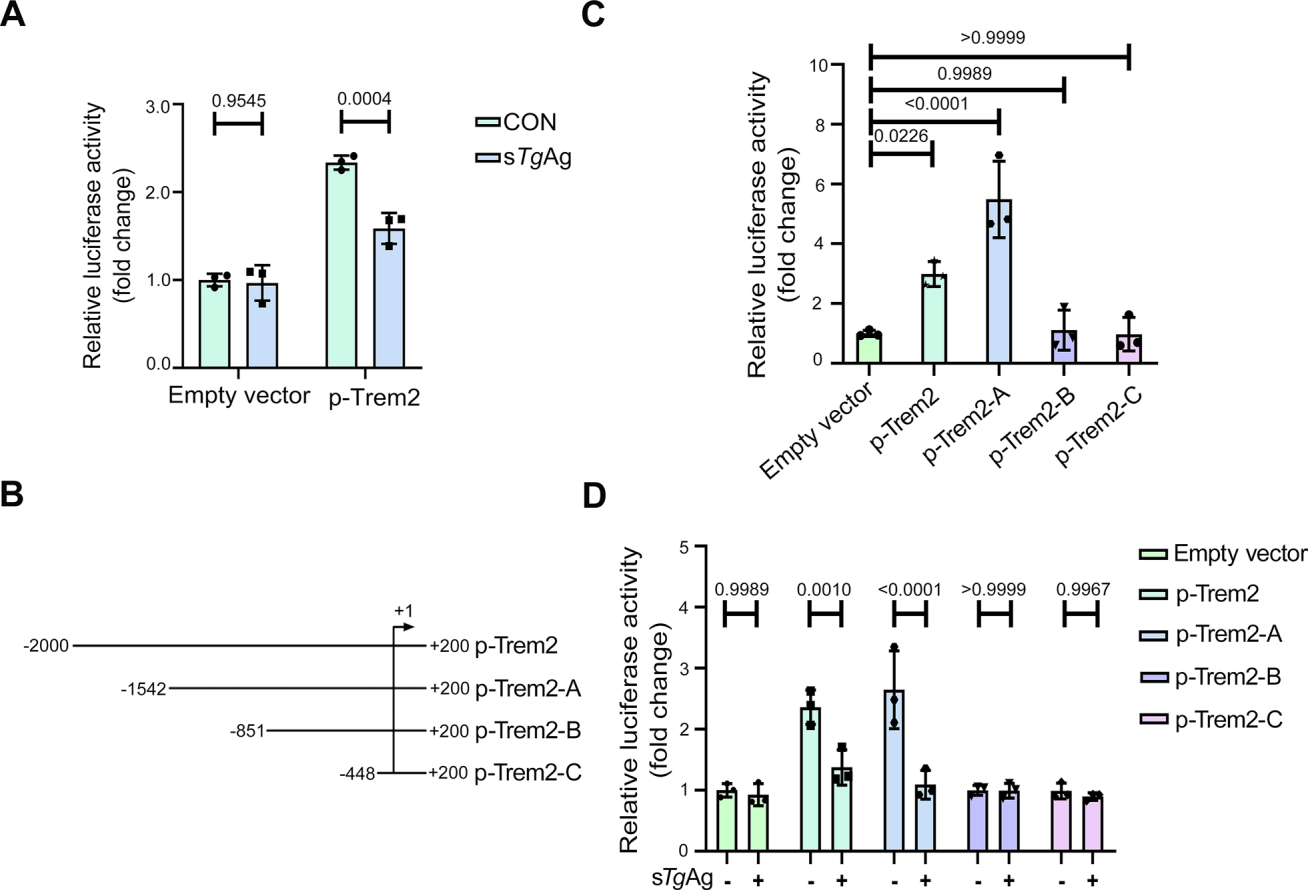
The activity of the *Trem2* promoter is inhibited by soluble *T. gondii* antigens. **A** The luciferase reporter gene vectors incorporating the entire length of *Trem2* promoter (p-Trem2), along with the pRL-TK, were co-transfected into Raw264.7 cells for 24 h. After Raw264.7 cells were exposed to 5 µg/ml of s*Tg*Ag for 24 h, the luciferase activity was assessed. **B** Schematic plasmid construction containing truncated fragments of the *Trem2* promoter, designated as p-Trem2-A (−1542 to +200), p-Trem2-B (−851 to +200), and p-Trem2-C (−448 to +200). **C** Luciferase activity was assessed at 24 h post-transfection after co-transfecting the recombinant plasmids with pRL-TK into Raw264.7 cells. **D** The recombinant plasmids and pRL-TK were co-transfected into Raw264.7 cells for 24 h, which were then exposed to 5 µg/ml of s*Tg*Ag, and luciferase activity was assessed at 24 h post-stimulation. (mean ± SD; *n* = 3 independent experiments; two-way ANOVA with Sidak’s multiple comparisons test (**A**, **D**); one-way ANOVA with Tukey’s multiple comparisons test (**C**). s*Tg*Ag, soluble *T. gondii* antigens; CON, the untreated control group

Gene promoters, which are key regions for transcription initiation, determine the binding site of RNA polymerase and the initiation of transcription, thereby affecting the level of gene expression [31, 32]. The core promoter is the smallest region that directs accurate transcription initiation. To identify the core promoter that can regulate the activity of the *Trem2* promoter, we constructed recombinant plasmids that encompass the full length of the* Trem2 *promoter (p-Trem2) and co-transfected them into cells along with the *Renilla* luciferase plasmids. Subsequently, we stimulated the cells with s*Tg*Ag (5 µg/ml) for 24 h and observed a considerable decrease in *Trem2* promoter activity, compared with the control group (Fig. 2A).

To explore the active region of the *Trem2* promoter, we then constructed the recombinant plasmids containing truncated fragments of the *Trem2* promoter region, named p-Trem2-A (−1542 to +200), p-Trem2-B (−851 to +200) and p-Trem2-C (−448 to +200), as shown in Fig. 2B. We then assessed the promoter activity by transfecting these recombinant plasmids into Raw264.7 cells. Notably, while the full-length *Trem2* promoter exhibited relatively high promoter activity, the most significant enhancement was observed when truncated to the −1542 to +200 region. However, further shortening the promoter length to −851 to +200 or −448 to +200 contributed to a loss of promoter activity. These results imply that the regions between −1542 and −851 are the core promoter regions that can enhance *Trem2* promoter activity (Fig. 2C).

Furthermore, upon stimulation with s*Tg*Ag, we assessed the luciferase activity of the various promoter fragments and found that the promoter activity of p-Trem2-A (−1542 to +200) was significantly decreased. In contrast, no substantial difference was observed in *Trem2* promoter activity between p-Trem2-B, p-Trem2-C, and unstimulated groups (Fig. 2D). This suggests that s*Tg*Ag, by targeting the *Trem2* promoter region (−1542 to −851), significantly downregulated the promoter activity of *Trem2* in Raw264.7 cells, thereby negatively regulating the promoter activity of *Trem2*.

### ATF3 binds to the −1542 to −851 region of the *Trem2* promoter

**Fig. 3:**
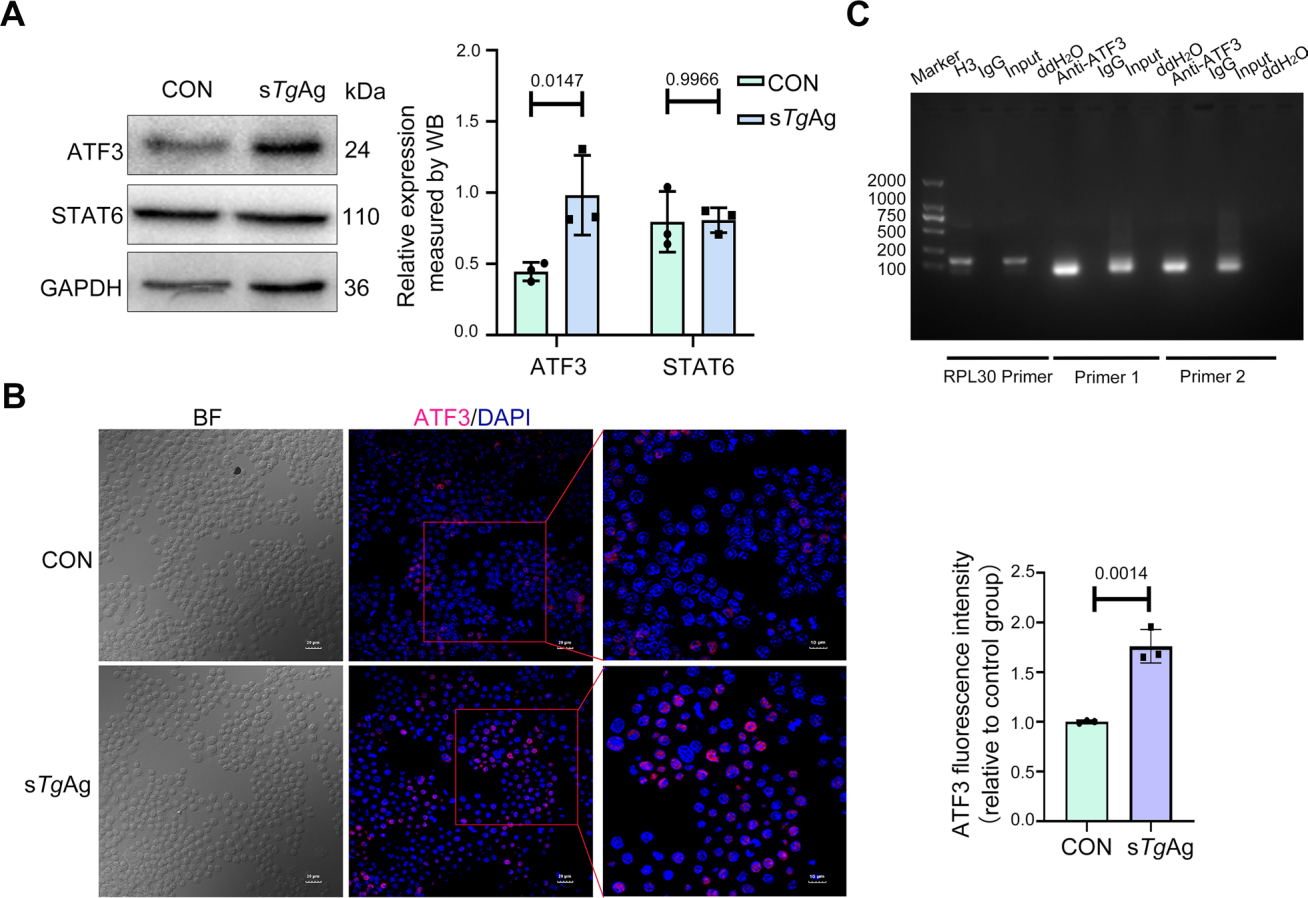
ATF3 binds to the −1542 to −851 region of the *Trem2* promoter. **A** Raw264.7 cells were exposed to 5 µg/ml of s*Tg*Ag for 48 h and screened by western blotting to identify transcription factors that can bind to the *Trem2* promoter. Analysis was performed using Image J. **B** Immunostaining and quantification of ATF3 (red) was conducted following stimulation of Raw264.7 cells with 5 µg/ml of s*Tg*Ag for 48 h. Scale bar: 20 μm. **C** ChIP analysis was assayed to confirm that the putative ATF3 transcription factor can bind to the *Trem2* promoter. RPL30 Primer served as a positive control group. Data are shown as the mean ± SD. A two-tailed unpaired Student’s *t*-test was used (**A**, **B**); *n* = 3 independent experiments; s*Tg*Ag, soluble *T. gondii* antigens; CON, the untreated control group. Quantifications normalized to GAPDH for western blot analysis

We sought to identify the transcription factors that regulate the active region of the *Trem2* promoter, which is located between the positions −1542 and −851. To achieve this, we utilized two bioinformatics platforms, PROMO and JASPAR, to predict potential transcription factors that could bind to this region. The analysis suggests that transcription factors such as ATF3 and STAT6, may act within the region. Subsequently, we observed an upregulation of ATF3 expression following stimulation with the s*Tg*Ag, as confirmed by protein immunoblotting, whereas STAT6 expression remained unaffected (Fig. 3A). Experiments with immunofluorescence analysis following stimulation of Raw264.7 cells with s*Tg*Ag further corroborated the increase in ATF3 expression (Fig. 3B). This provided further evidence supporting the role of s*Tg*Ag in enhancing ATF3 levels. To confirm the binding of ATF3 to the* Trem2* promoter, we used primers aimed at two anticipated binding sites and performed ChIP assays. We found that ATF3 can bind to the critical regulatory region of the *Trem2* promoter (Fig. 3C). In summary, our findings indicate that s*Tg*Ag promotes the expression of ATF3, which then binds to the *Trem2 *promoter −1542 to −851 region.

### Soluble *T. gondii* antigens repress *Trem2* promoter activity in an ATF3-dependent manner

**Fig. 4:**
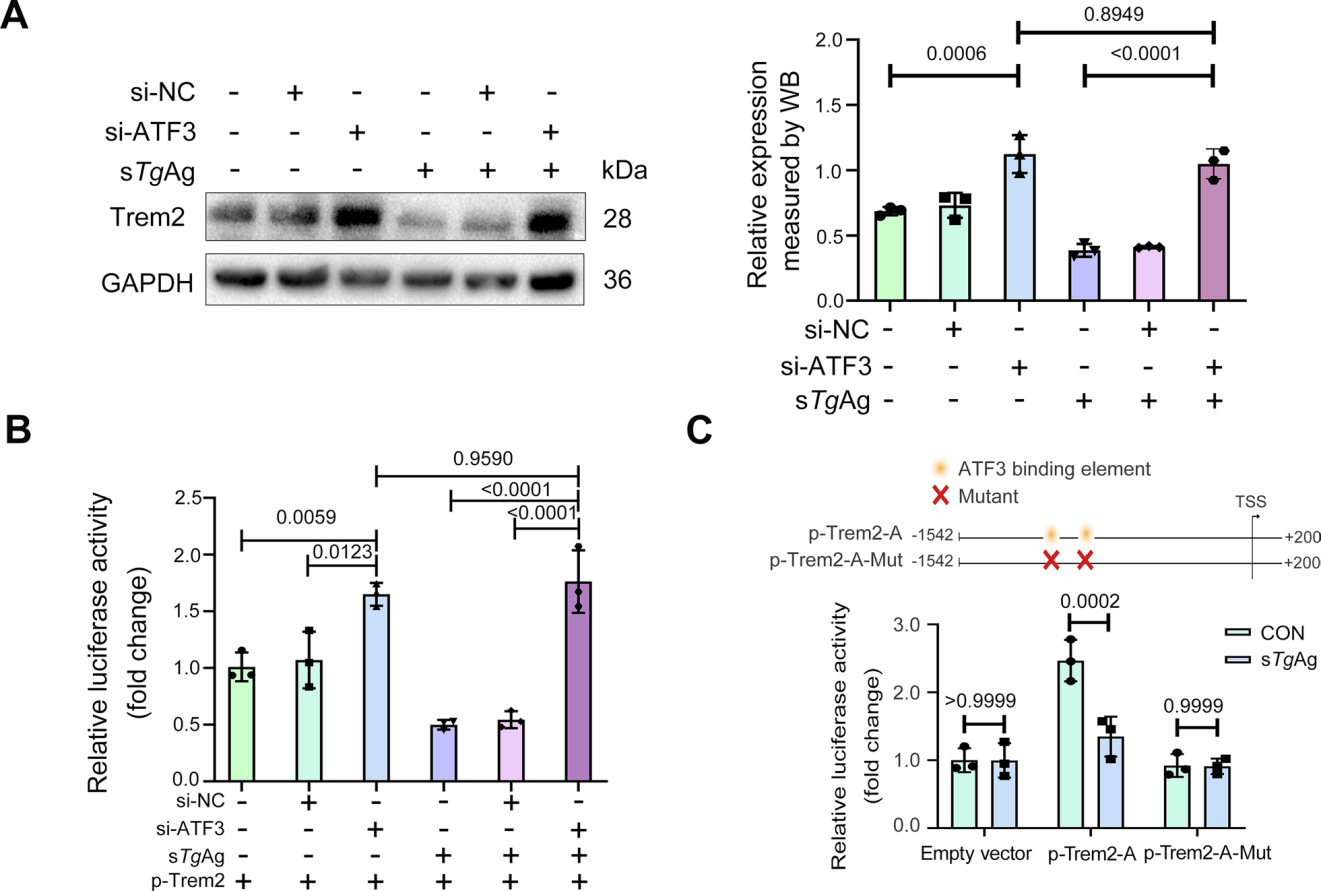
Soluble *T. gondii* antigens repress *Trem2* promoter activity in an ATF3-dependent manner. **A** *ATF3* was knocked down by transfecting Raw264.7 cells with small interfering *ATF3* (20 nM) and the negative control siNC (20 nM). After 6 h of transfection, cells were exposed to s*Tg*Ag at 5 µg/ml for 48 h. Trem2 expression was assessed using western blotting, with analysis performed with Image J. **B** The luciferase reporter gene vector containing the *Trem2* promoter (p-Trem2) was co-transfected into Raw264.7 cells along with pRL-TK after the transfection with siATF3 (20 nM) or siNC (20 nM) in Raw264.7 cells. Following 6 h of transfection, luciferase activity in Raw264.7 cells was measured after 24 h of incubation with 5 µg/ml of s*Tg*Ag. (**C**) Schematic representation of ATF3 binding site-specific mutations at −1364 to −1354 and −1134 to −1124 in the *Trem2* promoter sequence are shown. The p-Trem2-A (−1542 to +200) and p-Trem2-A-MUT luciferase reporter gene vectors were transfected into Raw264.7 cells for 24 h, which were subsequently exposed to 5 µg/ml of s*Tg*Ag, and luciferase activity was assessed after 24 h post-stimulation. A two-way ANOVA with Sidak’s multiple comparisons test was used (**A**, **B**, and **C**); *n* = 3 independent experiments; mean ± SD; TSS, transcription start site; s*Tg*Ag, soluble *T. gondii* antigens; CON, the untreated control group. Quantifications normalized to GAPDH for western blot analysis

To determine whether the transcription factor ATF3 regulates *Trem2* expression in macrophages treated with s*Tg*Ag, we transfected Raw264.7 cells with *ATF3* siRNA and performed protein immunoblotting assays after 48 h. Our observations showed a marked increase in Trem2 expression upon *ATF3* knockdown in Raw264.7 cells following stimulation with s*Tg*Ag (Fig. 4A). A luciferase reporter assay performed after stimulation of Raw264.7 cells with s*Tg*Ag further confirmed the increased *Trem2* promoter activity after ATF3 inhibition, consistent with our previous findings (Fig. 4B). Thus, *ATF3* knockdown can reverse the downregulation of Trem2 caused by s*Tg*Ag. We found that ATF3 can bind to the critical regulatory region of the *Trem2* promoter via ChIP assays. To further confirm the interaction between ATF3 and the *Trem2* promoter, we mutated the two ATF3 binding sites within the −1542 to −851 region of the *Trem2* promoter. Upon stimulation with s*Tg*Ag, we observed a decrease in dual-luciferase activity in Raw264.7 cells transfected with p-Trem2-A. However, no change was detected in cells transfected with p-Trem2-A-Mut (Fig. 4C). Consequently, our observations indicate that the transcription factor ATF3 can combine with the *Trem2* promoter and that ATF3 loses its regulatory influence on the *Trem2 *promoter activity once these binding sites are mutated. Hence, s*Tg*Ag can suppress Trem2 expression by upregulating the transcription factor ATF3, which binds to the *Trem2* promoter and thereby inhibits its activity.

## Discussion

This research elucidates the mechanism by which *T. gondii* suppresses* Trem2* promoter activity through ATF3-dependent transcriptional regulation. Trem2 serves as a master regulator of macrophage function and inflammatory modulation [33]. In the absence of Trem2, macrophages fail to effectively support the migration and proliferation of trophoblast cells and to express anti-inflammatory molecules in vitro [15]. This deficiency can exacerbate APOs caused by *T. gondii* infection. Our study reveals how s*Tg*Ag regulates the *Trem2* promoter through a novel mechanism distinct from known Trem2 signaling pathways. ATF3 was identified as the key transcription factor that binds to the *Trem2* promoter and inhibits its expression in response to s*Tg*Ag, providing new insight into Trem2’s transcriptional regulation. This inhibitory mechanism could represent a strategic maneuver by *T. gondii* to escape the host immune response.

dMφ at the maternal–fetal interface function as antigen-presenting cells during pregnancy, playing a crucial role in maintaining immune homeostasis and protecting the fetus from external pathogens through various molecular and cellular mechanisms [34, 35]. When the lysophosphatidic acid (LPA)–autophagy axis is defective, it can inhibit the expression of adhesion molecules on dMφ and impair M2 differentiation, thereby increasing the risk of abortion in early pregnancy [36]. Moreover, *T. gondii* infection significantly increases the ratio of dMφ in wildtype mice and further upregulates the expression of molecules such as CD80, CD86, iNOS, and cytokines such as TNF-α in dMφ. This leads to the impairment of the JAK2/STAT1 pathway and causes dMφ dysfunction [14]. Based on our previous findings, *T. gondii* infection modulates the Trem2/Syk/PI3K signaling axis to regulate dMφ functionality, thereby contributing to APOs [15]. These results indicate that dMφ dysfunction is involved in *T. gondii*-induced APOs.

*Trem2*, specifically expressed on macrophages, is a key functional molecule that controls metabolic homeostasis, promotes phagocytosis of bacteria, and inhibits inflammatory response [37–39]. Its role has also been studied in parasite diseases such as schistosomiasis [19]. Zhu et al. found that *Trem2* is critical for the M2 macrophage polarization [40]. In mice with *Trem2* deficiency, M2 macrophage markers, such as *Arg1* and *Ym1*, were downregulated in *Schistosoma japonicum* (*S. japonicum*)-infected liver tissues, yet Trem2 deficiency elevated the percentage of F4/80^+^CD86^+^ cells in peritoneal macrophages of infected mice. In a previous study, we found that in *T. gondii*-infected mouse placentas, M1-related markers (*iNOS* and *CD86*) were elevated in wildtype and Trem2^−/−^ mice, while M2-related markers (*Arg1* and *CD206*) were reduced, suggesting an elevated M1-polarization bias but a reduced M2-polarization bias. Furthermore, *Trem2* deficiency in mice aggravated M1-type bias in *T. gondii*-infected placentas [15]. In the context of parasite infection, *Trem2* promotes macrophage M2 polarization but suppresses M1 polarization.

ATF3 is the key transcriptional repressor of *Trem2* in *T. gondii* infection. It is well established that promoters can interact with multiple transcription factors, thereby regulating the expression of genes involved in macrophage polarization [41]. Unlike p53, which activates the *Trem2* promoter to enhance phagocytosis [42]. Our study utilized ChIP experiments and constructed recombinant plasmids containing serial truncation fragments of the* Trem2* promoter to demonstrate that the transcription factor ATF3 represses *Trem2* promoter activity by directly attaching to the *Trem2* promoter. Research using p53 knockout Raw264.7 cells further confirmed that LPS suppresses Trem2 expression through non-p53 pathways [42]. However, our findings indicate that s*Tg*Ag fails to suppress Trem2 expression or its promoter activity when ATF3 expression is inhibited or the ATF3 binding site on the *Trem2* promoter is mutated. Thus, s*Tg*Ag can inhibit *Trem2 *promoter activity in an ATF3-dependent manner. A recent study reported that miR-3473b suppresses Trem2 expression by binding to the 3′ UTR region of *Trem2* [43]. miR-3473b and the transcription factor ATF3 play different roles in regulating *Trem2* expression, with the transcription factor ATF3 influencing transcription by directly binding to the *Trem2* promoter.

ATF3 plays a dual role in regulating inflammatory responses [25]. This transcription factor has a wide array of properties that allow it to either repress or activate transcriptional functions, and in some studies, acts as a pure trans-activator [44, 45]. Several studies have shown that ATF3 serves as a positive regulatory molecule in the context of infections caused by Gram-positive bacteria, where it facilitates the infiltration of macrophages, and stimulates the production of pro-inflammatory cytokines [46, 47]. In contrast, ATF3 serves as a negative regulator during infections caused by Gram-negative bacteria, such as *Escherichia coli* (*E. coli*) and *Neisseria gonorrhoeae* (*N. gonorrhoeae*), by inhibiting the synthesis of inflammatory cytokines [48, 49]. Notably, we identified that ATF3—a known repressor of NF-κB-dependent transcription—significantly inhibits* Trem2* promoter activity [50]. In this study, once macrophages were stimulated with s*Tg*Ag, *Trem2* expression decreased, while ATF3 expression increased. Thus, in the context of *T. gondii* infection, ATF3 was identified as a repressor that regulates the promoter activity of *Trem2*.

## Conclusions

In summary, our study uncovers how *T. gondii* manipulates the ATF3–Trem2 axis to disrupt dMφ function and promote APOs. Critically, we identified that the transcription factor ATF3 binds to the *Trem2* promoter and negatively regulates its activity. This mechanism could potentially offer a novel therapeutic approach for addressing APOs triggered by *T. gondii* infection. Understanding the role of ATF3 in *T. gondii* infection is crucial, as it provides insights into how *T. gondii* manipulates host immune responses to facilitate its survival and replication.

## Supplementary Information

Additional file 1.

Additional file 2.

## Data Availability

Sequence data that support the findings of this study have been deposited in Mendeley Data at https://data.mendeley.com/datasets/rhwh8c758k/2.
