# Supplementary material for: ATF3-mediated inhibition of Trem2 by Toxoplasma gondii contributes to adverse pregnancy outcomes
Source: Parasit Vectors. 2025 Jul 1;18:245. doi: 10.1186/s13071-025-06894-w (PMC12210541; doi:10.1186/s13071-025-06894-w)
Supplement: Supplementary file 1 — Additional file 1. [file 13071_2025_6894_MOESM1_ESM.docx]

**supplementary Figures**

**Fig. S1.**


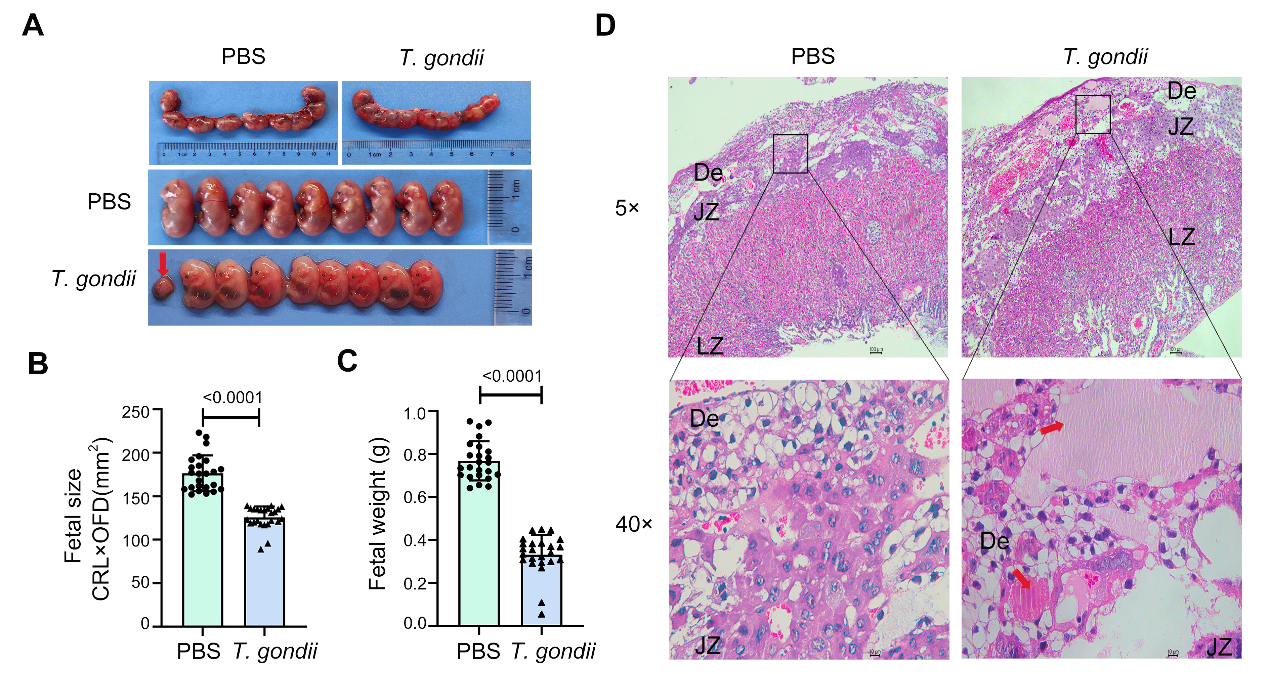


Trem2 expression is suppressed by *T. gondii* infection in mouse placenta. (A) The photographs illustrate representative images of the uterus and fetus with marked hemorrhagic necrosis of the placenta with fetal abortion (red arrow) in the *T. gondii-*infected pregnancy mice. (B and C) Fetal development is evaluated based on fetal size, calculated as crown-rump length (CRL) and occipito-frontal diameter (OFD), along with fetal weight. Every data point corresponds to individual fetuses (n = 6 mice). (D) Representative HE stained placental sections from *T. gondii*-infected pregnant mice demonstrated extensive hemorrhagic necrosis (indicated by red arrows). Histological examination revealed pathological changes across all placental compartments, including the labyrinth zone (LZ), decidua zone (De), and junctional zone (JZ). (two-tailed unpaired Student′s *t*-test for all data; mean ± SD.)
